# Assessing Drought Tolerance in a Large Number of Upland Cotton Plants (*Gossypium hirsutum* L.) under Different Irrigation Regimes at the Seedling Stage

**DOI:** 10.3390/life13102067

**Published:** 2023-10-16

**Authors:** Sadettin Çelik

**Affiliations:** Department of Forestry, Genç Vocational School, University of Bingol, Bingol 12500, Turkey; sadettincelik@bingol.edu.tr

**Keywords:** drought, cotton, root, shoot, tolerance, water scarcity

## Abstract

The cotton plant is important since it provides raw materials for various industry branches. Even though cotton is generally drought-tolerant, it is affected negatively by long-term drought stress. The trial was conducted according to the applied experimental design as a completely randomized design (CRD) with three replications to determine a panel of 93 cotton genotypes’ genotypic responses against drought under controlled conditions in 2022. All genotypes were watered with 80 mL^−1^ of water (100% irrigation, field capacity) until three true leaves appeared, and then water stress was applied at a limited irrigation of 75% (60 mL^−1^), 50% (40 mL^−1^), and 25% (20 mL^−1^) of the field capacity. After the trial terminated at 52 days, the cv. G56, G44, G5, and G86 in RL; G1, G56, G44, G86, G51, and G88 in RFW; advanced line G5, followed by the cv. G56, advanced line G44, G75, and the cv. G90 in RDW; G44, followed by G86, the cv. G56, and elite lines G13 and G5 in NLRs were observed as drought-tolerant genotypes, respectively, while G35, G15, G26, G67, and G56 in SL; G15, G52, G60, G31, and G68 in SFW; G35, G52, G57, G41, and G60 in SDW show the highest drought tolerance means, respectively. In conclusion, the commercial varieties with high means in roots, namely G86, G56, G88, and G90, and the genotypes G67, G20, G60, and G57 showing tolerance in shoots, are suggested to be potential parent plants for developing cotton varieties resistant to drought. Using the cultivars found tolerant in the current study as parents in a drought-tolerant variety development marker-assisted selection (MAS) plant breeding program will increase the chance of success in reaching the target after genetic diversity analyses are performed. On the other hand, it is highly recommended to continue the plant breeding program with the G44, G30, G19, G1, G5, G75, G35, G15, G52, G29, and G76 genotypes, which show high tolerance in both root and shoot systems.

## 1. Introduction

Cotton (*Gossypium* spp.) provides raw materials to many industries with its various economic features, such as natural fiber for the textile sector, cotton seed for the oil sector, and pulp for the livestock sector [1,2,3,4]. This industrial plant, which directly and indirectly affects the livelihood of many people around the world, is drought-tolerant and is cultivated by about 30 countries, primarily China, India, the USA, Pakistan, and Brazil due to their warm climate [5,6]. However, intensive cultivation also leads to the emergence of various biotic diseases and pests and abiotic stress factors. Abiotic stress factors, such as drought and salinity, prevent the water intake of the cotton, and biotic stress factors, such as Verticillium and Fusarium, cause xylem vessels to be clogged and the plant to not receive sufficient water, which causes a decrease in the turgor pressure that provides the verticality of the plants and causes the leaves to wilt and the fruits to be produced [7]. Drought means that plants suffer from water shortages for a long period on a global scale because of the greenhouse gas effect as a result of human and non-human activities. Drought is one of the most important abiotic stress factors that cause 73% of product loss in cotton [8]. In particular, in the USA, which is one of the leaders in world cotton production, the production loss due to drought stress in the last 50 years is approximately 67% [9], while it is 34% in Pakistan [10].

Although cotton has a higher tolerance to drought compared to other cultivated industrial field crops, when it is exposed to long-term drought stress, undesirable effects such as a decrease in yield, lower biomass, and stem weight, a slowdown in plant development, a decrease in fiber quality, and small bolls may occur [11]. Drought adversely affects *Gossypium* spp., along with other plants, by limiting plant height, leaf weight, the number of nodes, transpiration rate, photosynthesis rate, and stomatal conductivity [11,12,13,14,15]. In addition, when it is exposed to long-term drought stress, it closes its stomata, rolls its leaves, tends to have osmotic regulation, and tries to reach water/moisture in the depths of the soil [16], and the rate of photosynthesis is reduced [17]. 

A great deal of research is being conducted to understand the physiological, morphological, and metabolic reactions of plants against drought in roots [18,19]. Under drought stress, the roots of the plants tend to go deeper and aim to reach underground water, and these behaviors of the roots also play an important role in activating other mechanisms related to drought [20]. Anjum et al. [21] indicated that a strong root system plays a very critical role in physiological functions, carbohydrate storage, uptaking, and the absorption of water and nutrients from the soil. In the first stage of drought, plants slow down the elongation of the stem and develop the root system to reach deeper water [22]. 

The responses of the cotton plant to stress factors vary during different stages of development. The seedling stage is a period of high sensitivity to environmental conditions for many plant species, including cotton [23]. The seedling stage is not only adversely affected by biotic stress factors but also negatively affected by abiotic stress factors, such as drought [24] and salinity [25]. Brand et al. [26] discovered that low temperatures at the seedling stage in cotton under chilling conditions are strongly associated with low temperatures of the seedling stage diseases such as dumping off, which causes root deterioration. Low temperatures at the seedling stage of cotton have a big effect on the damage thresholds of pathogens such as Rhizoctonia and Pythium [27]. Furthermore, seedling emergence rates and development under controlled conditions can determine the sensitivity of varieties to cold weather [28]. When the cotton seeds emerged and were exposed to sub-optimal temperatures in late spring, seedling growth was adversely affected, and significant decreases were observed in yield [29]. When soil moisture tension generally exceeds 30–50 centibars, drought stress occurs, depending on soil type [30].

To understand the root system responses under water scarcity conditions: a short-term drought during the seedling period increased the cotton roots ability to reach deep water but decreased the diameter of the roots [31]. It is revealed that the cotton roots were affected by drought more than the root distribution [32]. Insufficient soil moisture reduces the elongation of the roots [33,34], and a shortened root system is developed approximately 42–70 days after the germination of the cotton [32]. Root growth is a critical process to be grown under sustainable temperatures [35,36]. Zahid et al. [37] stated that 22–30 °C is an optimal temperature for cotton root and shoot growth and an optimal root/shoot (R/S) ratio, but a temperature between 32 and 40 °C will restrict the distribution and growth of the root/shoot system. A similar finding was declared by Koevoets et al. [38] that the optimal temperature promotes an increase in the root/shoot ratio (R/S ratio); nevertheless, a temperature above optimal temperature will decrease water absorption and plant nutrients in the cotton plant root system, and as a results, the root system get weaker against drought [39]. A decrease in soil water content by evaporation significantly reduces root activities, but hydraulic lifting and transpiration by roots can increase groundwater levels to levels close to the soil surface (to 0–40 cm soil depth) [40]. 

Various studies have been conducted to understand cotton plants’ genotypic responses against drought. Studies such as developing cultivars that are tolerant to drought through crossbreeding [41], hormonal regulation against drought and salinity stress [42], understanding the drought tolerance mechanism of cotton and integrating it into variety development breeding programs [43], drought stress generation and a determination of tolerant genotypes in the cotton plant using Polyethylene Glycol (PEG) [44], determining drought-stress-related traits in the seedling period [45], strategies for coping with drought stress in cotton and their application to crop improvement programs [45], increasing the drought tolerance of cotton genotypes by exposing them to salinity during the seedling period [46], understanding the allelic genetic diversity of drought-resistant cotton genotypes [47], and identifying cultivars carrying drought genes using Trait-assisted selection (MAS) in cotton [48] have been carried out. 

The research conducted shows that the seedling stage is the period during which plant reactions can be observed the best [49]. This study aims to investigate the drought-tolerant level of some advanced lines and some genetic stock cultivars of the cotton (*Gossypium hirsutum* L.) species under artificial drought stress with limited irrigation methods during the seedling stage. In this study, unlike many studies, not only are cultivars evaluated under drought stress conditions, but also advanced lines’ genetic potential against drought is investigated.

## 2. Materials and Methods

### 2.1. Plant Materials

All of the 93 cotton genetic materials used in the experiment are a panel of both accessions from advanced lines and commercial varieties of *Gossypium hirsutum* L. with allotetraploid (2n = 4x = 52) chromosomes set with AD_1_ genome groups (Table 1). The genotypes were provided by the East Mediterranean Transitional Zone Agricultural Research Institute, East Mediterranean Agricultural Research Institute, GAP International Agricultural Research and Training Institute, SET seed company STARseed, MAY BASF, and Progen Inc., seed companies. The commercial cultivars’ properties, such as cotton seed yield, resistance to some technological properties, and climate requirements, are known. Due to continuous segregation, the economic properties of advanced breeding lines are still not clear, so this experiment provides information about the individual properties of advanced lines, including reactions to drought.

### 2.2. Method

The pattern of the trial is the applied experimental design as a completely randomized design (CRD) with 3 replications. The experiment was conducted at Bingol University, Genc Vocational School (Coordinates: 38 °442’58” N and 40 °32′11” E), climate chamber. The soil used is prepared with peat, perlite, and soil in a ratio of 3:1:1. All three components were sterilized with an autoclave at 121 °C for 15 min and transferred to 200 mL plastic pots. Triangular holes were made at the bottom of the plastic pots for the excess water to be drained. four seeds were sowed in each pot at a depth of about 2 cm, and potting soil was brought to the field capacity. For control purposes, STV373 (G86), BA119 (G56, White gold), and TEX (G93) varieties adapted to the Southeastern Anatolia Region, where the experiment was established, were used. In the experiment, three pots were used for each genotype as the 3 replications. The soil temperature was raised to 15 °C for seeds to germinate, and since the experiment was established in winter, the cotton seeds, which are likely to be dormant, were kept in a hot water bath device at 65 °C for 15–30 min before sowing to break the dormancy. Then, the ambient temperature was adjusted to be between 28 and 38 °C, and the humidity was between 57 and 67% [50]. 

LED lights were adjusted to provide daylight at 2.500 lux for 14–15 h a day, and each pot was given 0.2 g of pure nitrogen-containing urea fertilizer for effective growth 14 days after planting. From the starting point of the experiment, cotton genotypes were irrigated with 100% irrigation every 10 days until true leaves bloomed. All genotypes were fully watered with 80 mL^-1^ of water (100% irrigation, field capacity) until three true leaves appeared. As soon as all the genotypes produced true leaves, the limited irrigation was started. Then, water stress was applied at limited irrigations of 75% (60 mL^−1^), 50% (40 mL^−1^), and 25% (20 mL^−1^) of the field capacity. The experiment was terminated on the 52nd day.

The cotton plants were taken out of their pots intact, the soil was washed using tap water without damaging the roots, and parameters such as the Root Length (RL), Root fresh weight (RFW), Root dry weight (RDW), Shoot length (SL), Shoot fresh weight (SFW), Shoot dry weight (SDW) and the number of lateral roots (NLRs) of one plant from each replication genotype were measured. RL was calculated by a direct measurement of fresh taproots, RFW by direct weighting of taproots, RDW was measured after drying in the oven for 24 hours at 60 °C, NLRs by direct counting of roots before drying, SL by measuring with a ruler, SFW by calculation with a scale with 0.5 mg precision, and SDW was measured after drying in the oven for 24 hours at 60 °C [50] 

### 2.3. Data Analysis

An analysis of variance (ANOVA) using data from these drought-related parameters was carried out using the statistical software JUMP 17.0 (JMP^®^, Version <17>. SAS Institute Inc., Cary, NC, USA, 1989–2021) [51]. The frequency charts were created by Microsoft Office Excel 2016 ver. The correlation diagram, cluster analysis, principal component analysis (PCA) analysis, and charts were obtained via JMP 17.00 ver. [51]. The Scatter plot charts were created using Minitab 19 [52], and the significance of the difference between the means of the parameters was investigated (*p* < 0.05). The least significant difference (LSD) was used to compare the means. 

## 3. Results

### 3.1. Analysis of Variance (ANOVA)

In the experiment, the RL, RFW, RDW, NLRs, SL, SFW, and SDW of the cotton germplasm panel were measured. As a result of the statistical analysis, it was observed that there was a highly significant difference between the means for RL, RFW, RDW, NLRs, SL, and SFW (*p* < 0.01). The statistical analysis conducted at a 95% confidence level (*p* < 0.05) revealed that, according to the mean squares, there is a significant difference among the genotypic means in terms of the shoot dry weight trait. Table 2 indicates the significance of the population mean differences for each trait.

### 3.2. Genotypic Performance under Drought

**Figure 1:**
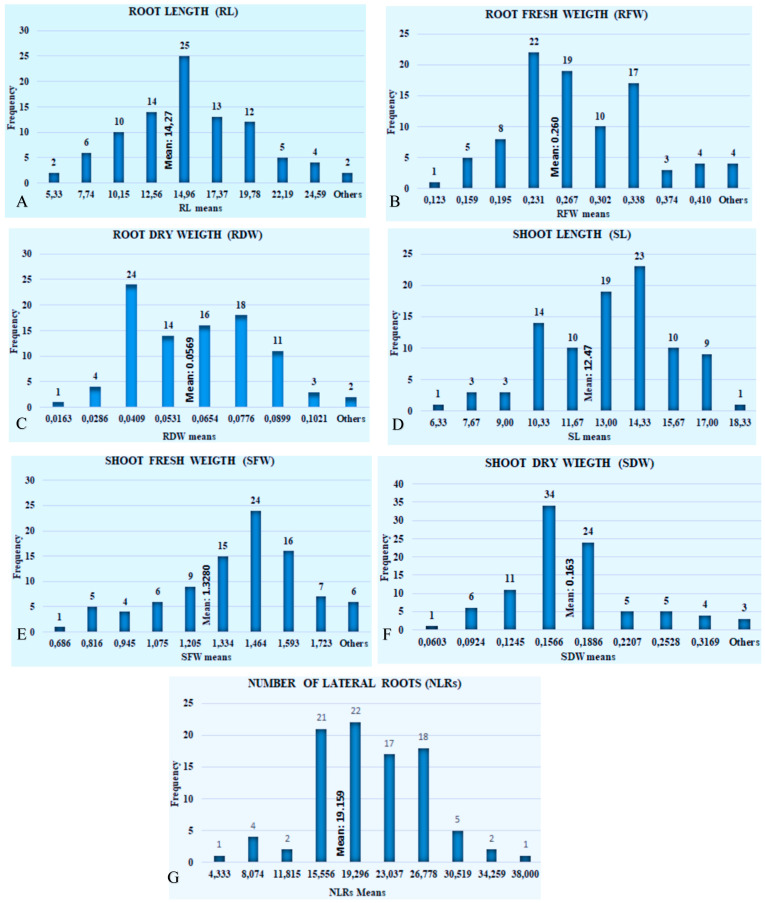
(**A**) Rooth length (RL), (**B**) root fresh weight (RFW), (**C**) root dry weight, (**D**) shoot length (SL), (**E**) shoot fresh weight (SFW), (**F**) shoot dry weight (SDW), and (**G**) number of lateral roots (NLRs) Frequency charts.

In this study, the genotypes with the longest roots (the ones with the highest tolerance against drought) were G56 (27 cm) and G44 (25,6), followed by the GG5 and G86 cultivars with 24.3 and 23.6 cm root lengths. G76 and G41 had the shortest RL (5.3 cm), followed by the G35 and G14 genotypes with a 5.7 cm root length (Supplementary Table S1). The mean RL of the population was 14.27 cm (Figure 1A). Figure 1A shows the mean distribution frequency of the genotypes regarding the root length. The developed root system turns in the direction where soil moisture is intense and solves the water problem of the plant, and the roots disperse deeply and to the sides to enable the plant to hold onto the soil more strongly under harsh weather conditions (phenotypic observation). 

The mean RFW of the entire population in the study was 0.26 g. The lowest root fresh weight was 0.123 g, and the RFW recorded in the G76 and G41 genotypes was 0.136 g. The advanced genotypes were followed by G35 (0.140 g), and the highest root fresh weight was recorded in the G1 (0.446 g) advanced line, followed by the cv. G56 (0.427 g), G44 (0.424 g), commercial cultivar G86 (0.411 g), G51 (0.397 g), and G88 (0.384 g) (Supplementary Table S1). According to Figure 1B, the RW–frequency histogram graph, the number of genotypes with a mean root weight above the mean of the whole population (0.260 g) is 57 (Supplementary Table S1). This may be an indication that most of the individuals in the population can activate their tolerance mechanisms against drought stress. 

Root dry weight (RDW) is also one of the phenotypic traits used to see the reactions of the cotton against drought. Accordingly, the mean RDW of the population, consisting of 93 genotypes, was 0.06 g. The advanced genotype G5 recorded the highest RDW value (0.127 g), followed by the cv. G56 (0.112 g), the advanced breeding line G44 (0.099 g), G75 (0.092 g), and the cv. G90 (0.091), and this value was followed by the G85 (0.088 g) and G7 (0.085 g) breeding genotypes. It is possible to say that the drought resistance genes show themselves in the phenotype of the G5, G56, and G44 cultivars. The control cultivar G56, which is well adapted to the Southeastern Anatolia and Mediterranean regions of Turkey, where cotton cultivation is most common, and shows tolerance above the general mean, is used as a control variety in the research (Supplementary Table S1). In Figure 1C, concerning the RDW values, the mean of the genotypes was 0.0569 g. There were 50 genotypes with means above the overall mean and 43 genotypes with means below the overall mean. The genotype with the highest frequency was 24. 

Accordingly, the mean total shoot length (SL) of the genotypes was 12.47 cm. G35 (18.33 cm) had the longest SL, and it was followed by the G15-G29 (16.67 cm) and G76 (16.33 cm) breeding genotypes, respectively. The commercial cultivar G65 (6.33 cm) showed the lowest SL means, followed by the elite lines G72-G32 (7.33 cm) and the local cotton variety G22 (7.33) (Supplementary Table S1). Regarding the SL (Shoot length) trait, 31 genotypes had means below the overall mean, and 62 genotypes had means above the overall mean (Figure 1D). 

The mean SFW of 93 cotton genotypes was 1.3280 grams. The highest SFW values were recorded in the following genotypes: the advanced line G15 (1.853 g), G52 (1.815 g), and the commercial variety G60 (1.805 g), respectively. These were followed by G31 (1.752 g), G41 (1.736 g), G47 (1.724 g), G14 (1.700 g), and the commercial local variety G68 (1.691 g). The lowest SFW value was measured in the advanced genotype G83 (0.686 g), followed by G2 (0.716 g), G75 (0.739 g), and G91 (0.740 g), respectively (Supplementary Table S1). Mostly of the cotton genotypes showed the expected responses under water scarcity. For another drought-related trait, SFW (shoot fresh weight), 24 genotypes had the highest means, while 68 genotypes had means above the population mean of 1.3280 g (Figure 1E).

The SDW mean of all the genotypes was recorded as 0.163 g. The highest SDW value was recorded in G35 (0.349 g) and the lowest in the G65 (0.060 g) genotype. While commercial cultivars such as G57 (0.337 g), G60 (0.294 g), and G66 (0.289 g) showed high mean SDW values, the advanced lines G52 (0.345 g) and G41 (0.314) showed very high SDW values. The second lowest SDW value was found for the commercial varieties G91 (0.078 g) and G4 (0.078 g) and G20 (0.078 g), followed by G22 (0.081 g) and the cv. G90 (0.087 g) (Supplementary Table S1). In the case of SDW (shoot dry weight), 34 genotypes had the highest frequency, and the majority of the population had SDW values below the population mean of 0.163 (Figure 1G).

In the current study, the G44 (38) RIL genotype had the greatest number of lateral roots (NLRs), and it was followed by G86 (33), the commercial cv. G56 (32), and the advanced lines G13 (30), G5 (29.33), and G49 (29). The mean of number of lateral roots of all the genotypes was 19.16 (Supplementary Table S1). Regarding the NLRs (number of lateral roots) characteristic, 21 genotypes had a mean of 15.556, 22 genotypes had a mean of 19.037, and 26.778 was the mean for 18 genotypes. The population NLR mean was 19.159, with 28 genotypes having means below it and 68 genotypes having means above it (Figure 1G).

**Figure 2:**
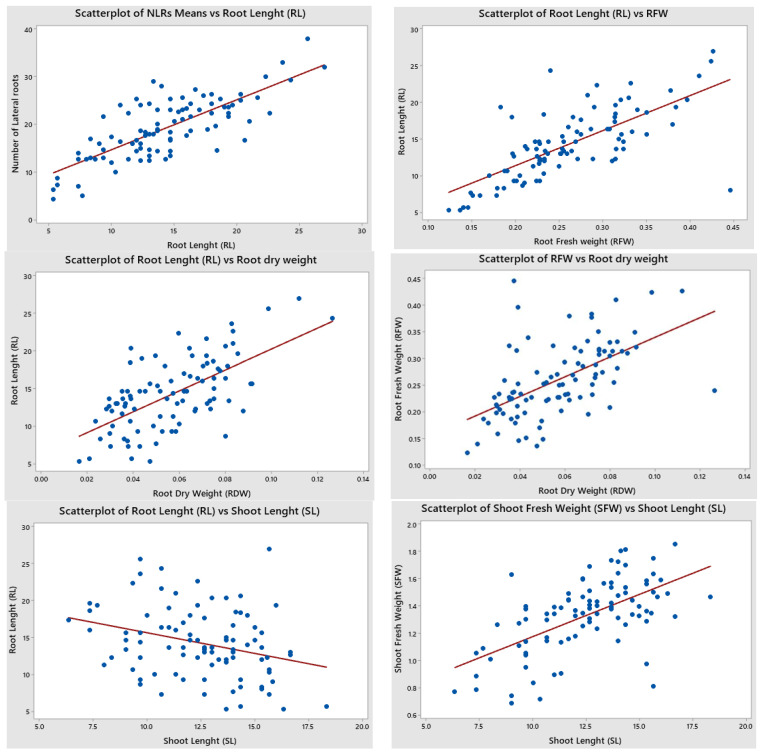
Scatter (XY) plot analysis of drought morphological traits using Minitab 19 [52] and correlation diagram in JMP 17.00 [51]. version.

The scatter plot analysis was carried out to determine the relationship and relationship type between every two variables of drought indicators. Yi [53] emphasized that the closer they get to the linear line, the more the relationship between parameters increases. According to the Figure 2 scatter plots and correlation diagram, while the strongest positive correlation is seen between RL and RFW (0.823), followed by NLRs and RL (0.759), RFW and NLRs (0.679), RFW–RDW (0.642), RL–RDW (0.568), and NLRs–RDW (0.494), the strongest negative correlation took place between SL–RDW (−0.446), followed by RFW–SL (−0.327), NLRs–SL (−0.318), NLRs–SFW (−0.219), and SDW–RFW (−0.143) (Table 3, Figure 2). Similar correlations between the root–shoot traits of the cotton plant were identified by [54].

A principal component analysis (PCA) is a powerful multivariate statistical technique widely used for identifying patterns of data, expressing the data, and highlighting their similarities and dissimilarities [55]. A PCA simplifies complex datasets by transforming them into a new coordinate system, where the data’s variance is maximized along the principal components. This dimensionality reduction retains most of the relevant information while eliminating redundant or noisy features, thus aiding in data compression and visualization [56].

**Figure 3:**
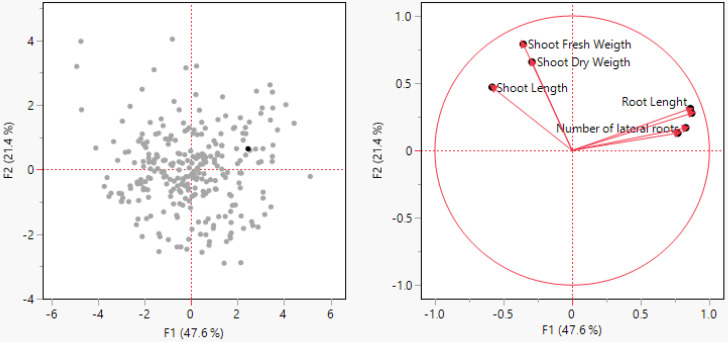
Correlation circle obtained by principal component analysis (PCA) of the variables factor space of F1 and F2 using JMP 17.00 ver. [51] statistical analysis software.

The graph (Figure 3) is the loading plot from the PCA analysis. The results of the PCA are given in Figure 3. The PCA was conducted based on the morphological drought parameters, and in the correlation circle, the first axes (F1) and the second axes (F2) represent the discriminant function analysis (DFA), respectively, 47.6% and 21.4% of the variable. Among the drought measurement parameters of the cotton plant’s aboveground portion, the strongest positive correlation was found between shoot dry weight (SDW) and shoot fresh weight (SFW), while shoot length (SL) showed a positive correlation with both SFW and SDW (Figure 3). Accordingly, as the SL increases, it appears that the SFW and SDW values increase positively. 

In terms of the underground drought measurement morphological parameters, a very close positive correlation between them is observed. The strongest positive correlation was found between root length (RL) and root fresh weight (RFW), while a significant positive correlation was also found between the number of lateral roots (NLRs) and root dry weight (RDW). Generally, there is a negative correlation between aboveground drought traits and underground traits in cotton plants. This situation can be explained by the increase in the aboveground parts being hindered due to strengthening the roots of cotton plants under water stress, transferring photosynthesis products from the aboveground parts to the roots to draw water from the soil more effectively (Figure 3).

**Figure 4:**
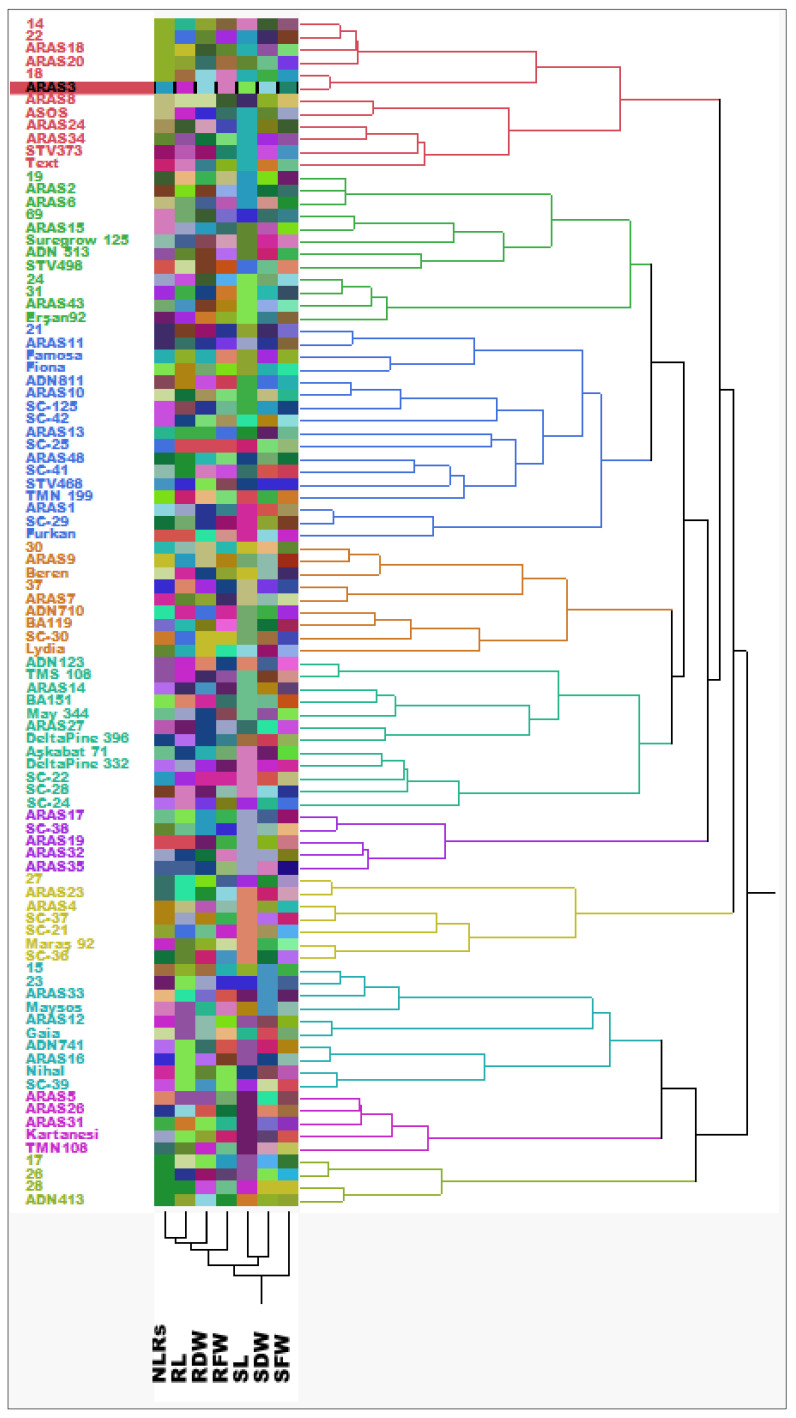
Cluster analysis based on genotypes genotypic responses under drought stress using JMP 17.00 ver. [51] statistical analysis software.

In Figure 4, generally, the most tolerant genotypes to drought are clustered at the top, while the most sensitive genotypes are clustered at the bottom. Based on tolerance–susceptible properties, ten clusters were obtained, with five being major and the other five being minor. The majority of the genotypes, in response to drought stress, exhibited different reactions in various organs, and a clear distinction was not observed (Figure 4).

The genotypic reactions of cotton genotypes under drought stress have been correlated and clustered based on their sensitivity and tolerance. Simultaneously, shoot traits and root traits have also been clustered according to their relationships with each other (Figure 4).

Accordingly, in root traits, the closest relationship was observed between the NLRs and RL, while in shoot traits, the closest relationship was observed between the SL and SDW. Although the root traits and shoot traits clustered together, a significant correlation was observed between them. The SFW, in particular, was found to be associated with all the other traits. The shoot fresh weight (SFW) may be concluded to have a very close relationship with all the other organs of the cotton plant due to its possession of chlorophyll pigments that enable photosynthesis and its production of glucose through the photosynthetic pathway.

## 4. Discussion 

The responses of plant roots under drought stress have become the focus of researchers in recent years [57,58]. The first plant organ that is exposed to water stress is the roots, and by transmitting this stress to the rest of the plant, it goes through morphological, physiological, and metabolic changes, and the plant starts to activate its mechanisms to cope with the water stress [18]. Root length (RL) growth has a great impact on the uptake of nutrients and root system architecture [59,60]. It is a current trend in plant breeding to select genotypes with the longest roots for drought tolerance [9,61]. It was seen in this study that several advanced breeding lines had a high tolerance to drought in some drought measurement parameters. The cv. G56 had the longest RL (27 cm), followed by G44 (25.67 cm), G5 (24.33 cm), and G86 (23.67 cm) in this study (Supplementary Table S1). Similar results were found by [50], which reported that taproots had a root length between 47.9 and 86.6 cm under full irrigation and between 48.3 and 82.5 cm under restricted irrigation in their experiment. The higher root length means of [50] compared to our results are believed to be dependent on the experimental conditions, trial materials, origins of the plant varieties used, amount of water used, and duration of the experiment. In the study, although the humidity and temperature in the greenhouse can increase excessively during the day, the amount of soil mixture used was parallel to our study, and a longer taproot system was observed. 

Research has shown that immersing roots in depths (root length) where the moisture content is high can compensate for the moisture losses that occur through evapotranspiration [62]. In the current study, the G76 and G41 advanced lines produced a 5.33 cm RL as the lowest RL, followed by G35, G14 (5.7), G79, G73, and G7 (7.3 cm) (Supplementary Table S1). Our results are similar to [63], with the RL trait varying between 17.10 cm and 30.80 cm. Similar root length (RL) values were obtained by Pawar and Veena [64], with the highest root length at 12.72, 12.17, and 12.88 cm and the lowest at 9.8, 9.85, and 10.5 cm with the PEG 6000 drought application. At the same time, Pawar and Veena [64] revealed that the root length increased when the PEG 6000 application was increased by 10% and decreased after that level. This situation can be explained by the plant root tolerance mechanism of the plant: the plant sends more photosynthesis products to the root in order to strengthen the roots and uptake more water from the soil [65]. In a study, Zahid et al. [66] found a highly significant correlation between genotypes and watering, and they found the root length to be 5.161 cm. According to the current study, with mean root length of 27 cm, 5.161 cm is highly low, and the irrigation period, the amount of water given in each irrigation, soil mixture, infiltration and germination ability, and speed can be shown as the reasons why it is found to be lower than our study. Wang et al. [67] reported that the distribution of photosynthetic products in the roots of beans increased under drought stress, which did not last long, but the photosynthetic products in the production organs decreased significantly. It was observed that the plant tries to strengthen its root system under long-lasting drought stress, thereby severely reducing its yield and economic added value as a result of giving all its weight to the roots. Studies reported that a situation similar to the growth period of wheat can be observed in cotton: moderate water stress causes photosynthetic products to be transported to the roots by vascular bundles and has a similar positive effect on buds and bolls [68]. It was reported that moderate water stress during the grain-filling period of the wheat plant increases the transport of photosynthetic products to the roots, and this provides a significant benefit to the wheat, as the wheat will have developed roots and thus take more moisture and therefore more nutrients from the soil [69]. 

A higher root weight enables the uptake of more plant nutrients from the soil and plays a key role in vegetative and generative development [70]. In the current study, the RFW trait values changed between 0.123 g (G76) and 0.446 g (G1), with a population mean of 0.260 g. The genotypes that were tolerant according to the root fresh weight trait were the cv. G56 (0.427 g), G44 (0.424 g), the commercial variety G86 (0.411 g), G51 (0.397 g), and G47 (0.148 g), respectively (Supplementary Table S1). Our findings are similar to Iqbal et al. [71], Mvula et al. [63], and Jaafar et al. [72]. Similarly, Shah et al. [73] obtained a root weight ranging from 0.049 to 0.155 g under water stress in a trial conducted under normal and water-stressed conditions for 52 days. In a similar study, Akbar and Hussain [74] obtained fresh root weights ranging from 0.222 g to 0.394 g under different drought stresses. In Figure 1B, it is observed that 22 genotypes have a mean RFW of 0.267, which is slightly higher than the population mean. The fact that 57 genotypes produce a mean higher than the population suggests genetic diversity within the population, with some genotypes exhibiting superior root growth in terms of fresh weight. The genotypic mean for the RDW is 14.27 (Figure 1B). The research showed that there is a positive correlation between root weight and drought and that the genotypes with quantitatively higher root weights had a higher tolerance to drought stress than the others. While the specific context or objectives of the study are not provided, this trait likely relates to the ability of genotypes to allocate resources to root growth.

Under long-term drought stress, the root dry weight increased and the leaf area decreased in Chinese spinach [75]. In the current study of drought response measurement root trait, root dry weight, the highest value was recorded in the advanced line G5 (0.127 g), followed by the control variety G56 (0.112 g), G44 (0.099 g), G75 (0.092 g), and control variety G90 (0.091 g), while lowest RDW values were recorded in the advanced line G76 (0.016 g), followed by genotypes G35 (0.021 g), G20 (0.024 g), G61 (0.026 g), G70 (0.028 g), G59-G15 (0.029 g), and G67 (0.030 g) (Supplementary Table S1). The RDW varied from 0.79 g to 1.18 g [63]. Istiqomah et al. (2021) [70] found a very significant difference between the applications in terms of dry root weight or total dry weight in corn plants. Similarly, in their study investigating the responses of the germination stage root drought trait to drought in cotton, Fathi-Sadabadi et al. [54] obtained dry root weights ranging from 0.00342 to 0.00685 g. In Figure 1C, the genotypes exhibit a wide range of RDW values, with 50 genotypes having means above the overall mean and 43 below. This variation could have practical implications for breeding programs, as it suggests the presence of genotypes with different root biomass production potentials. The variation in genotypic means for RDW (Figure 1C) indicates that some genotypes are more efficient at producing dry root biomass than others. 

As the importance of NLRs traits observed in this current study indicates, the number and development of lateral roots are important indicators of drought tolerance. The development of the root system, which allows plants to stand, hold on to the soil, give yield, and grow and develop, plays an active role in the fight against agricultural drought. Wang et al. [76] stated that many previous studies related to the crop–root system improvement suggest that increasing the number of lateral roots (NLRs) could characterize plant development and increase crop yield. Lateral root numbers and development are regulated by many diverse hormones and their interactions [77,78]. Lateral root initiation (LRI) and development is coordinated by many genes. In *Arabidopsis thaliana*, there are more than seven genes associated with lateral roots [79,80]. The population mean of the number of lateral roots (NLRs) changed from 5 to 38. The advanced line G44 (38) showed the highest NLRs, and this value was followed by G86 (33), the cv. (control variety) G56 (32), G13 (30), and G5 (29.33). The lowest NLR values ranged from 5 to 10. In the current study, the lowest NLRs were recorded in G47 (5), followed by G76 (6.33), and G79 (7) (Supplementary Table S1). Similarly, the NLR value showed variability between 29.67 and 47.10 [63]. McMichael et al. [81] obtained similar NLR values ranging from 4 to 50 at 30 °C within 7 days after germination. The reason for the higher LRN values may be associated with not applying drought stress in the first 7 days and plenty of water and plant nutrients. In a study with 120 exotic cotton genotypes provided, the NLRs mean changed from 3.5, 17.5, 25.97, and 49.75, respectively [81]. The genotypic means for NLRs vary significantly (Figure 1G), with 28 genotypes below the population mean and 68 genotypes above it. This trait might be related to drought tolerance or stress response, and the observed variation suggests potential candidates for further research or breeding programs. Lateral roots and numbers play a key role in plants reaching and absorbing water [82] and uptaking plant nutrients such as phosphorus [83]. Hund et al. [84,85]. Gallardo et al. [86] reported that enough water in soil accelerates root development, and Luo et al. [87] reported that moderate water stress in the middle and upper layers of the soil strengthens the root system in the early stage of cotton plant development and contributes significantly to roots reaching deep waters. The number, development, and growth of lateral roots are critical for the development and yield of field crops [88,89]. In another study, Forde and Lorenzo [90] stated that the lateral roots, which are important in plants with a taproot system, initially act as taproots and take water and nutrients dissolved in water. To increase tolerance to drought stress, it was seen that researchers should focus on the development of genotypes of lateral root systems. It was seen that the drought-resistance RIL genotypes G44 (38), G86 (33), and G56 (32) varieties, which have good adaptation in the Southeast Anatolia region of Turkey, where cotton is mainly cultivated, develop better lateral root systems under drought stress. Even though the current study breeding lines have developed good taproot systems, they may not show high resistance against high temperatures compared to commercial cultivars adapted in warm regions. It has been put forward that lateral root numbers can have positive effects of vigorous growth under low temperatures in maize plants. Considering the studies in the literature, it is seen that the number of lateral roots is a drought trait that increases the tolerance of plants against drought stress. 

Shoot length (SL) can be used as a criterion for the selection of drought-tolerant plants [91,92]. As a result of this study, it has been observed that shoot length (SL) during the seedling stage is one of the organs most affected by drought in cotton, and the population mean obtained was 12.47, while the genotype means ranged from 18.33 cm (G35) to 6.33 cm (G91) (Supplementary Table S1). The shoot length averages ranging from 35.20 cm to 43.37 cm, as reported by [63], resemble our findings. During the initial period that drought stress is observed, it has been noted that the shoot length remains constant, but in the advanced stages of drought, the shoot length starts to increase. This situation can be explained by the fact that the plant absorbs more water from the soil, leading to increased photosynthesis and the production of C_6_H_12_O_6_ (glucose). Similarly, Iqbal et al. [71] reported that drought during the seedling stage negatively affects both the shoot and root morphology of cotton, reducing the shoot length by 29%. A study indicated that drought stress causes a significant reduction in shoot elongation, and the percentage of decreasing SL ranged from 97.14 to 35.1 [66]. A reduction in the shoot or in the root length may be caused by an imbalance of water [93]. Genotypes with a mean SL of 12.47 (Figure 1D) show significant variability, with 31 genotypes below the population mean and 62 above it. This trait may be important in the context of plant height and overall shoot development, which can impact crop yield and plant architecture. 

For shoot fresh weight (SFW) drought traits, an advanced line, G15, has the weight with a 1.853 g value, followed by G52 (1.815 g) and the commercial variety G60 (1.805 g), respectively. (Supplementary Table S1). Similar SFW trait results were obtained by Akbar and Hussain [74], Lund and Elsayed [94], and Iftikhar et al. [95]. In Figure 1E, 24 genotypes exhibit the highest means for SFW, with 68 genotypes exceeding the population mean of 13.280. This suggests that there are genotypes with a higher capacity for shoot growth and biomass production, which could be advantageous in terms of yield potential.

In Figure 1G, the majority of genotypes have SDW values below the population mean. This indicates that, in general, genotypes tend to have less dry shoot biomass compared to the population mean. However, the presence of genotypes with higher SDW values could be of interest for further study or breeding efforts. The best SDW value was recorded in G35 (0.349 g) and the lowest in the G65 (0.060 g) genotype. While commercial cultivars such as G57 (0.337 g), G60 (0.294 g) had lower SDW values. (Supplementary Table S1). In a study, Ahmad et al. [96] obtained similar results while investigating the root–shoot traits responses under water deficiency. The genotype G35, which shows the highest value in shoot dry weight (SDW), also exhibits the highest value in the shoot length (SL) parameter as a drought trait. Genotypes G22 and G91 have low means in the SL, SFW, and SDW parameters, indicating their low tolerance to drought stress (Supplementary Table S1). Basal et al. [50] highlighted traits such as SFW and SDW, due to their ease of measurement and reliability, that could be used as selection criteria in measuring drought tolerance. The genotype G35 has emerged as the most tolerant genotype in both traits, with a mean SL of 18.33 cm and SDW of 0.349 g. Meanwhile, genotype G15 registers the second-highest mean SL, and the same genotype gave the highest SFW mean (Supplementary Table S1). These values indicate that an increase in SL does not always correspond to the highest SFW; however, there is a positive correlation between an increase in SL and SFW. A similar situation is observed in the tolerant genotype G31 for the SL and SFW traits. On the contrary, the genotype G91 has the lowest SL value. Generally, genotypes with higher SL means have higher SFW means, and genotypes with lower SL means have lower SFW values

In the experiment, among plants that shade each other, those receiving less light exhibit higher shoot length but lower shoot fresh and shoot dry weights compared to plants receiving full light. This situation can be explained by the hypothesis that as the amount of light increases, the plant enhances its photosynthetic rate and consequently increases its biomass. Based on the conducted phenotypic observations, it has been observed that under greenhouse conditions, cotton genotypes exhibit shorter shoot lengths compared to the same duration of laboratory trials. However, it is evident that the experiment established in greenhouse conditions has significantly longer root lengths and a higher lateral root count compared to the laboratory drought trial. This suggests that when the plant experiences actual water stress, it strengthens its roots and engages in coping mechanisms to deal with water scarcity. This response aims to minimize the impact of drought and indicates the activation of defense mechanisms to mitigate the effects of water stress.

The provided information presents valuable insights into the performance of various genotypes concerning SFW and SDW traits, as well as their comparison to control genotypes. Genotypes G60, G52, and G41 stand out for their high mean in both SFW and SDW, indicating their potential as superior performers in terms of biomass production. A noteworthy observation is made with genotype G52, which exhibits a positive correlation between SFW and SDW. As the SFW increases, the SDW also increases proportionally. This correlation suggests that as these plants put on more fresh weight, they also accumulate more dry weight, indicating an efficient conversion of water and nutrients into biomass. This is a desirable trait in agriculture as it signifies robust growth and resource utilization efficiency (Supplementary Table S1). However, the genotype G60 presents an interesting contrast. While it boasts the highest SFW value among the genotypes, it ranks sixth in SDW. This discrepancy suggests that in the case of G60, an increase in fresh weight does not necessarily lead to a corresponding increase in dry weight. This could be due to various factors, such as differences in water content or the allocation of resources within the plant. Further investigation is warranted to understand this specific genotype’s growth pattern better. (Supplementary Table S1). 

## 5. Conclusions

In this current study, the genotypes G44 and G56 exhibit common high values in all four drought traits (NLRs, RL, RFW, and RDW). The genotypes G13 and G5 share high values in NLRs and RL, while the genotype G5 coincides in terms of NLRs, RL, and RDW. The genotypes G1, G56, G51, and G88 show the highest values in RFW, and the genotypes G5, G56, G44, G75, and G90 consistently yield the highest means in the RDW traits. The genotype G35 shows the highest mean values in both SL and SDW; the genotype G15 shares high values in both SL and SFW; and the genotypes G41, G52, and G60 have high values in both the SFW and SDW parameters, demonstrating high drought tolerance. Using the tolerant cultivars found in the current study as parents in drought-tolerant variety development in the marker-assisted selection (MAS) plant breeding program will increase the success of reaching the target after genetic diversity analyses are performed. It is also possible for the genes responsible for this tolerance to transfer from the donor plants to the recipient plants through hybridization. 

## Figures and Tables

**Table 1:** Upland cotton genotypes properties and origins.

Code	Genotype Name	Genome Group	Species	Origin	Code	Genotype Name	Genome Group	Species	Origin	Code	Genotype Name	Genome Group	Species	Origin
G1	14	AD1	*G. hirsutum* L.	Türkey	G32	ARAS10	AD1	*G. hirsutum* L.	Türkey	G63	Fiona	AD1	*G. hirsutum* L.	Türkey
G2	15	AD1	*G. hirsutum* L.	Türkey	G33	ARAS11	AD1	*G. hirsutum* L.	Türkey	G64	Furkan	AD1	*G. hirsutum* L.	Türkey
G3	17	AD1	*G. hirsutum* L.	Türkey	G34	ARAS12	AD1	*G. hirsutum* L.	Türkey	G65	Gaia	AD1	*G. hirsutum* L.	Türkey
G4	18	AD1	*G. hirsutum* L.	Türkey	G35	ARAS13	AD1	*G. hirsutum* L.	Türkey	G66	Kartanesi	AD1	*G. hirsutum* L.	Türkey
G5	19	AD1	*G. hirsutum* L.	Türkey	G36	ARAS14	AD1	*G. hirsutum* L.	Türkey	G67	Lydia	AD1	*G. hirsutum* L.	Türkey
G6	21	AD1	*G. hirsutum* L.	Türkey	G37	ARAS15	AD1	*G. hirsutum* L.	Türkey	G68	Maraş 92	AD1	*G. hirsutum* L.	Türkey
G7	22	AD1	*G. hirsutum* L.	Türkey	G38	ARAS16	AD1	*G. hirsutum* L.	Türkey	G69	May 344	AD1	*G. hirsutum* L.	Türkey
G8	23	AD1	*G. hirsutum* L.	Türkey	G39	ARAS17	AD1	*G. hirsutum* L.	Türkey	G70	Maysos	AD1	*G. hirsutum* L.	India
G9	24	AD1	*G. hirsutum* L.	Türkey	G40	ARAS18	AD1	*G. hirsutum* L.	Türkey	G71	Nihal	AD1	*G. hirsutum* L.	Türkey
G10	26	AD1	*G. hirsutum* L.	Türkey	G41	ARAS19	AD1	*G. hirsutum* L.	Türkey	G72	SC-125	AD1	*G. hirsutum* L.	Türkey
G11	27	AD1	*G. hirsutum* L.	Türkey	G42	ARAS20	AD1	*G. hirsutum* L.	Türkey	G73	SC-21	AD1	*G. hirsutum* L.	Türkey
G12	28	AD1	*G. hirsutum* L.	Türkey	G43	ARAS23	AD1	*G. hirsutum* L.	Türkey	G74	SC-22	AD1	*G. hirsutum* L.	Türkey
G13	30	AD1	*G. hirsutum* L.	Türkey	G44	ARAS24	AD1	*G. hirsutum* L.	Türkey	G75	SC-24	AD1	*G. hirsutum* L.	Türkey
G14	31	AD1	*G. hirsutum* L.	Türkey	G45	ARAS26	AD1	*G. hirsutum* L.	Türkey	G76	SC-25	AD1	*G. hirsutum* L.	Türkey
G15	37	AD1	*G. hirsutum* L.	Türkey	G46	ARAS27	AD1	*G. hirsutum* L.	Türkey	G77	SC-28	AD1	*G. hirsutum* L.	Türkey
G16	69	AD1	*G. hirsutum* L.	Türkey	G47	ARAS31	AD1	*G. hirsutum* L.	Türkey	G78	SC-29	AD1	*G. hirsutum* L.	Türkey
G17	ADN 513	AD1	*G. hirsutum* L.	Türkey	G48	ARAS32	AD1	*G. hirsutum* L.	Türkey	G79	SC-30	AD1	*G. hirsutum* L.	Türkey
G18	ADN123	AD1	*G. hirsutum* L.	Türkey	G49	ARAS33	AD1	*G. hirsutum* L.	Türkey	G80	SC-36	AD1	*G. hirsutum* L.	Türkey
G19	ADN413	AD1	*G. hirsutum* L.	Türkey	G50	ARAS34	AD1	*G. hirsutum* L.	Türkey	G81	SC-37	AD1	*G. hirsutum* L.	Türkey
G20	ADN710	AD1	*G. hirsutum* L.	Türkey	G51	ARAS35	AD1	*G. hirsutum* L.	Türkey	G82	SC-38	AD1	*G. hirsutum* L.	Türkey
G21	ADN741	AD1	*G. hirsutum* L.	Türkey	G52	ARAS43	AD1	*G. hirsutum* L.	Türkey	G83	SC-39	AD1	*G. hirsutum* L.	Türkey
G22	ADN811	AD1	*G. hirsutum* L.	Türkey	G53	ARAS48	AD1	*G. hirsutum* L.	Türkey	G84	SC-41	AD1	*G. hirsutum* L.	Türkey
G23	ARAS1	AD1	*G. hirsutum* L.	Türkey	G54	ASOS	AD1	*G. hirsutum* L.	Türkey	G85	SC-42	AD1	*G. hirsutum* L.	Türkey
G24	ARAS2	AD1	*G. hirsutum* L.	Türkey	G55	Aşkabat 71	AD1	*G. hirsutum* L.	Turkmenistan	G86	STV373	AD1	*G. hirsutum* L.	USA
G25	ARAS3	AD1	*G. hirsutum* L.	Türkey	G56	BA119	AD1	*G. hirsutum* L.	Türkey	G87	STV468	AD1	*G. hirsutum* L.	USA
G26	ARAS4	AD1	*G. hirsutum* L.	Türkey	G57	BA151	AD1	*G. hirsutum* L.	Türkey	G88	STV498	AD1	*G. hirsutum* L.	USA
G27	ARAS5	AD1	*G. hirsutum* L.	Türkey	G58	Beren	AD1	*G. hirsutum* L.	Türkey	G89	SG 125	AD1	*G. hirsutum* L.	USA
G28	ARAS6	AD1	*G. hirsutum* L.	Türkey	G59	DeltaPine 332	AD1	*G. hirsutum* L.	USA	G90	Text	AD1	*G. hirsutum* L.	USA
G29	ARAS7	AD1	*G. hirsutum* L.	Türkey	G60	DeltaPine 396	AD1	*G. hirsutum* L.	USA	G91	TMN 199	AD1	*G. hirsutum* L.	Türkey
G30	ARAS8	AD1	*G. hirsutum* L.	Türkey	G61	Erşan92	AD1	*G. hirsutum* L.	Türkiye	G92	TMN108	AD1	*G. hirsutum* L.	Türkey
G31	ARAS9	AD1	*G. hirsutum* L.	Türkey	G62	Famosa	AD1	*G. hirsutum* L.	USA	G93	TMS 108	AD1	*G. hirsutum* L.	Türkey

**Table 2:** One-way ANOVA of drought traits.

Mean Squares (Water-Stressed)								
Source	df	NLRS	RL	RFW	RDW	SL	SFW	SDW
Genotypes	92	122.330 **	65.75 **	0.0147 **	0.0014 **	19.27 **	0.2067 **	0.0099 *
Replication	2	21.552	12.14	0.0054	0.0003	16.48	0.2593	0.0076
Error	184	16.711	12.44	0.0035	0.0004	6.29	0.0590	0.0071

* Significant at *p* = 0.05 level, ** Significant at *p* = 0.01 level. NLRs: number of lateral roots, RL: root length, RFW: root fresh weight, RDW: root dry weight, SL: shoot Length, SFW: shoot fresh weight, SDW: shoot dry weight.

**Table 3:** Correlation between studied traits.

Traits	NLRs	RL	RFW	RDW	SL	SFW	SDW
Number of lateral roots	****	0.7591	0.6795	0.4942	−0.3188	−0.2193	−0.1242
Root length	0.7591	****	0.8232	0.5681	−0.2684	−0.1105	−0.0932
Root fresh weight	0.6795	0.8232	****	0.6420	−0.3274	−0.0866	−0.1433
Root dry weight	0.4942	0.5681	0.6420	****	−0.4465	−0.1015	−0.1284
Shoot length	−0.3188	−0.2684	−0.3274	−0.4465	****	0.4774	0.2154
Shoot fresh weight	−0.2193	−0.1105	−0.0866	−0.1015	0.4774	****	0.4189
Shoot dry weight	−0.1242	−0.0932	−0.1433	−0.1284	0.2154	0.4189	****

## Supplementary Materials

The following supporting information can be downloaded at https://www.mdpi.com/article/10.3390/life13102067/s1, Table S1: Drought morphological markers mean in Upland cotton (*Gossypium hirsutum* L.) at the seedling stage.
