# Supplementary material for: Assessing Drought Tolerance in a Large Number of Upland Cotton Plants (Gossypium hirsutum L.) under Different Irrigation Regimes at the Seedling Stage
Source: Life (Basel). 2023 Oct 16;13(10):2067. doi: 10.3390/life13102067 (PMC10608038; doi:10.3390/life13102067)
Supplement: Supplementary file 1 [file life-13-02067-s001.zip › life-2638306-supplementary.pdf]

Supplementary Table S1. Drought morphological markers mean in Upland cotton (*Gossypium hirsutum* L.) at the seedling stage

CODE	Genotypes	NLRs	RL	RFW	RDW	SL	SFW	SDW
G1	14	12.67	8	0.446	0.037	15.33	1.585	0.183
G2	15	17.33	10	0.170	0.049	10.33	0.716	0.156
G3	17	22.33	19.3	0.182	0.050	12.33	1.596	0.253
G4	18	12.67	14.3	0.228	0.057	9.67	0.947	0.078
G5	19	29.33	24.3	0.240	0.127	10.67	1.142	0.171
G6	21	20.67	21.0	0.282	0.083	11.33	1.387	0.152
G7	22	12.67	7.3	0.151	0.043	10.67	1.341	0.152
G8	23	17.00	14.7	0.227	0.045	15.00	1.327	0.156
G9	24	16.67	20.7	0.330	0.080	14.33	1.263	0.150
G10	26	22.33	22.7	0.332	0.083	12.33	1.602	0.144
G11	27	18.00	13.3	0.264	0.073	9.00	1.630	0.155
G12	28	22.33	18.0	0.196	0.070	10.00	0.833	0.100
G13	30	30.00	22.3	0.293	0.060	9.33	1.111	0.180
G14	31	8.67	5.7	0.145	0.039	14.33	1.700	0.185
G15	37	18.33	12.7	0.198	0.029	16.67	1.853	0.228
G16	69	18.67	16.3	0.305	0.080	15.00	1.399	0.145
G17	ADN 513	14.67	13.0	0.252	0.072	13.00	1.433	0.153
G18	ADN123	14.67	9.3	0.198	0.042	12.67	1.510	0.165
G19	ADN413	22.33	11.3	0.250	0.057	8.00	1.008	0.108
G20	ADN710	16.33	10.7	0.187	0.024	15.67	0.809	0.078
G21	ADN741	23.00	14.7	0.255	0.052	12.33	1.250	0.153
G22	ADN811	23.33	16.0	0.333	0.070	7.33	0.885	0.081
G23	ARAS1	20.00	13.7	0.315	0.075	11.00	1.394	0.160
G24	ARAS2	25.67	21.7	0.378	0.072	10.67	1.168	0.162
G25	ARAS3	13.00	9.3	0.228	0.057	14.33	1.496	0.151
G26	ARAS4	19.00	17.7	0.275	0.077	12.67	1.381	0.124
G27	ARAS5	15.00	12.3	0.233	0.040	14.00	1.310	0.135
G28	ARAS6	21.67	16.3	0.286	0.068	10.67	1.298	0.301
G29	ARAS7	13.33	13.0	0.237	0.037	16.67	1.323	0.172
G30	ARAS8	21.67	19.3	0.290	0.066	16.00	1.591	0.108
G31	ARAS9	10.00	10.3	0.232	0.060	15.67	1.752	0.172
G32	ARAS10	24.00	19.7	0.314	0.085	7.33	0.784	0.154
G33	ARAS11	20.67	15.0	0.318	0.075	13.67	1.537	0.175
G34	ARAS12	12.33	12.3	0.271	0.074	12.33	1.465	0.159
G35	ARAS13	7.33	5.7	0.140	0.021	18.33	1.466	0.349
G36	ARAS14	23.00	17.0	0.380	0.062	11.67	1.441	0.149
G37	ARAS15	18.67	13.7	0.224	0.035	13.00	1.343	0.176
G38	ARAS16	18.33	14.7	0.269	0.063	12.33	1.347	0.127
G39	ARAS17	14.33	14.7	0.324	0.035	13.67	1.391	0.165
G40	ARAS18	12.67	8.7	0.208	0.080	9.67	1.395	0.131
G41	ARAS19	4.33	5.3	0.136	0.047	13.67	1.736	0.314
G42	ARAS20	12.67	11.3	0.220	0.052	13.00	1.402	0.125

G43	ARAS23	18.00	13.3	0.234	0.061	12.67	1.407	0.153
G44	ARAS24	38.00	25.7	0.424	0.099	9.67	1.376	0.249
G45	ARAS26	27.33	16.7	0.260	0.065	14.00	1.144	0.133
G46	ARAS27	28.00	14.0	0.211	0.039	14.67	1.336	0.135
G47	ARAS31	5.00	7.7	0.148	0.050	14.00	1.724	0.195
G48	ARAS32	16.67	12.0	0.228	0.067	13.67	1.423	0.137
G49	ARAS33	29.00	13.3	0.255	0.082	14.00	1.479	0.156
G50	ARAS34	16.00	12.3	0.314	0.067	9.67	1.054	0.094
G51	ARAS35	25.00	20.3	0.397	0.039	13.67	1.572	0.148
G52	ARAS43	19.67	18.3	0.232	0.072	14.33	1.815	0.345
G53	ARAS48	24.33	18.0	0.314	0.081	12.00	1.325	0.150
G54	ASOS	21.67	9.3	0.224	0.053	9.67	1.136	0.134
G55	Aşkabat 71	14.33	12.0	0.233	0.031	15.33	1.560	0.139
G56	BA119	32.00	27.0	0.427	0.112	15.67	1.634	0.162
G57	BA151	17.67	12.7	0.224	0.036	11.67	1.489	0.337
G58	Beren	24.00	10.7	0.190	0.039	9.33	1.272	0.172
G59	DeltaPine 332	23.00	13.7	0.213	0.029	15.33	0.972	0.104
G60	DeltaPine 396	14.51	18.4	0.315	0.039	14.12	1.805	0.294
G61	Erşan92	17.00	8.3	0.179	0.026	14.33	1.537	0.219
G62	Famosa	12.00	10.0	0.205	0.031	11.33	0.903	0.094
G63	Fiona	17.67	16.0	0.271	0.056	11.33	1.131	0.102
G64	Furkan	23.67	19.0	0.339	0.044	11.00	0.895	0.151
G65	Gaia	24.00	12.3	0.288	0.074	8.33	1.262	0.060
G66	Kartanesi	16.67	14.7	0.238	0.037	14.00	1.312	0.289
G67	Lydia	16.00	9.0	0.210	0.030	15.83	1.468	0.203
G68	Maraş 92	12.33	13.0	0.259	0.033	12.67	1.691	0.252
G69	May 344	14.33	13.7	0.253	0.039	11.67	1.157	0.131
G70	Maysos	18.67	12.3	0.228	0.028	15.57	1.347	0.156
G71	Nihal	13.33	14.7	0.222	0.043	12.00	1.365	0.159
G72	SC-125	25.33	18.7	0.351	0.075	7.33	1.052	0.110
G73	SC-21	14.00	7.3	0.178	0.038	12.67	1.435	0.204
G74	SC-22	13.00	8.3	0.187	0.036	15.33	1.289	0.160
G75	SC-24	23.00	15.7	0.322	0.092	9.00	0.739	0.091
G76	SC-25	6.33	5.3	0.123	0.016	16.33	1.492	0.205
G77	SC-28	25.67	15.7	0.275	0.047	15.33	1.362	0.151
G78	SC-29	24.33	16.3	0.308	0.075	11.00	1.341	0.124
G79	SC-30	7.00	7.3	0.159	0.030	15.67	1.499	0.141
G80	SC-36	24.33	13.0	0.251	0.059	12.67	1.308	0.162
G81	SC-37	19.00	13.7	0.324	0.055	12.67	1.284	0.158
G82	SC-38	16.00	11.7	0.227	0.035	13.67	1.375	0.172
G83	SC-39	25.33	14.7	0.222	0.062	9.00	0.686	0.161
G84	SC-41	26.33	18.0	0.266	0.054	14.67	1.443	0.160
G85	SC-42	25.33	12.0	0.310	0.088	13.33	1.568	0.149
G86	STV373	33.00	23.7	0.411	0.083	9.67	1.301	0.167
G87	STV468	22.67	15.3	0.253	0.050	12.00	1.179	0.146

G88	STV498	23.67	19.3	0.384	0.072	7.67	1.090	0.125
G89	Suregrow 125	26.33	20.3	0.321	0.064	13.00	1.231	0.112
G90	Text	21.00	15.7	0.350	0.091	9.67	1.039	0.087
G91	TMN 199	26.00	17.3	0.314	0.078	6.33	0.770	0.078
G92	TMN108	18.00	13.0	0.196	0.032	14.00	1.639	0.233
G93	TMS 108	14.67	9.3	0.201	0.058	11.67	1.444	0.170
	Means:	19.16	14.27	0.26	0.06	12.47	1.33	0.16

Abbreviations=Number of lateral roots (NLRs), Root length (RL), Rooth fresh weight (RFW), Root dry weight (RDW), Shoot length (SL), Shoot fresh weight (SFW), Shoot dry weight (SDW)
